# *Bacillus anthracis* TIR Domain-Containing Protein Localises to Cellular Microtubule Structures and Induces Autophagy

**DOI:** 10.1371/journal.pone.0158575

**Published:** 2016-07-08

**Authors:** Emil Carlsson, Joanne E. Thwaite, Dominic C. Jenner, Abigail M. Spear, Helen Flick-Smith, Helen S. Atkins, Bernadette Byrne, Jeak Ling Ding

**Affiliations:** 1 Department of Life Sciences, Imperial College London, London, United Kingdom; 2 Department of Biological Sciences, Faculty of Science, National University of Singapore, Singapore; 3 Defence Science and Technology Laboratory, Porton Down, Salisbury, United Kingdom; University of Cambridge, UNITED KINGDOM

## Abstract

Toll-like receptors (TLRs) recognise invading pathogens and mediate downstream immune signalling via Toll/IL-1 receptor (TIR) domains. TIR domain proteins (Tdps) have been identified in multiple pathogenic bacteria and have recently been implicated as negative regulators of host innate immune activation. A Tdp has been identified in *Bacillus anthracis*, the causative agent of anthrax. Here we present the first study of this protein, designated BaTdp. Recombinantly expressed and purified BaTdp TIR domain interacted with several human TIR domains, including that of the key TLR adaptor MyD88, although BaTdp expression in cultured HEK293 cells had no effect on TLR4- or TLR2- mediated immune activation. During expression in mammalian cells, BaTdp localised to microtubular networks and caused an increase in lipidated cytosolic microtubule-associated protein 1A/1B-light chain 3 (LC3), indicative of autophagosome formation. *In vivo* intra-nasal infection experiments in mice showed that a BaTdp knockout strain colonised host tissue faster with higher bacterial load within 4 days post-infection compared to the wild type *B*. *anthracis*. Taken together, these findings indicate that BaTdp does not play an immune suppressive role, but rather, its absence increases virulence. BaTdp present in wild type *B*. *anthracis* plausibly interact with the infected host cell, which undergoes autophagy in self-defence.

## Introduction

Toll-like receptors (TLRs) are a family of type I integral membrane proteins with key roles in the innate immune response, the first line of defence against invading pathogens [1]. The TLRs contain an extracellular domain comprised of a leucine-rich repeat linked via a single transmembrane region to an intracellular Toll/interleukin 1 receptor (TIR). Central to both the initiation and propagation of TLR signalling are heterotypic TIR-TIR interactions involving the TLRs and cytosolic adaptor proteins. There are four TIR containing TLR adaptor proteins involved in upregulation of the innate immune response, Myeloid differentiation factor 88 (MyD88), TIR domain containing adaptor protein inducing interferon β (TRIF), MyD88-adaptor like (MAL, also known as TIRAP) and TRIF-related adaptor molecule (TRAM) [2]. MAL and TRAM are bridging adaptors mediating recruitment of MyD88 and TRIF, respectively, to active TLRs, although both MyD88 and TRIF can interact directly with some TLRs [3]. This in turn is thought to cause association of other proteins crucial in TLR signalling, into a multi-protein complex called a Supramolecular Organizing Centre (SMOC) [4]. The SMOC propagates downstream signalling leading to activation of the NFκB transcription factor and thus, production of proinflammatory cytokines and type I interferons, central to the host response against infection. A fifth TIR domain containing protein, Sterile α and armadillo-motif containing protein (SARM) has been shown to be a negative regulator of the TLR system [5, 6]. SARM is likely to be a part of the normal homeostatic regulation of the TLR signalling system although its precise mechanism of action remains unclear [7]. SARM has also been shown to associate with cytoskeletal structures [8] and regulate microtubule stability via tubulin acetylation [9].

TIR domain proteins (Tdps) have also been identified in a range of microbes [10] including a number of pathogenic bacterial species [11–14]. Several of these proteins have roles in virulence [11–13] and there is substantial evidence that they are involved in subversion of the innate immune response [14–16]. In most cases it appears that the bacterial Tdp domains function to interfere with the heterotypic TIR-TIR interactions essential for initiation and propagation of the TLR signalling pathway [17]. To this end the bacterial Tdps appear to act as molecular mimics. This is illustrated by the fact that TIR domains present in bacterial Tdps have core structures very similar to those of mammalian TIR domains [15, 18]. For example, the structure of the TIR domain of *Brucella melitensis* TcpB shows root mean square deviation (RMSD) values of 2.5–3.0 Å for the TIR domain structures of human MyD88, MAL and TLR2 [15, 19]. The functionally important BB loop, named for connecting the strand βB and helix αB, adopts similar conformations in the two bacterial TIR domain structures solved, however this loop adopts markedly different conformations in the mammalian TIR protein structures [15]. The amino acid residues in the BB loop have been shown to play important roles in the normal functioning of the TLR signalling pathway [20–23] and also in the inhibitory function of bacterial Tdps [14, 16].

A Tdp has previously been identified in *Bacillus anthracis* [10], the causative agent of anthrax. Expression of the Tdp gene in *B*. *anthracis* is upregulated 2.3 fold in mouse macrophages between 1–2 h post-infection [24], a possible indication that the protein is functionally related to virulence. *B*. *anthracis* spores typically infect mammals via inhalation and are subsequently subjected to phagocytosis by macrophages whereupon they germinate. However, the mechanisms regulating intracellular development, and how the *B*. *anthracis* bacteria resist lysosomal degradation inside the cell, are not fully understood. Hu and colleagues have previously shown that cultured primary mouse macrophages efficiently kill both anthrax spores and vegetative bacteria within 4 h of infection [25], making the process behind initiation of infection *in vivo* unclear.

In light of previous research [17], we speculated that this protein (denoted BaTdp in this manuscript, equivalent to BA_4098 in *B*. *anthracis* Ames), may be involved in the evasion of the host immune response through negative regulation of the TLR signalling pathway. However, in addition to the production of inflammatory cytokines and chemokines, macrophages are known to utilise other mechanisms to combat bacterial infection, including the initiation of autophagy in order to maintain cellular homeostasis [26]. During this process, cytosolic components are wrapped into double membrane autophagosomes which later fuse with lysosomes in order to degrade the target, thus comprising an important cellular housekeeping tool. Interestingly, as this is a host defence mechanism, several pathogens have also evolved to utilise this pathway in order to establish a replicative niche and promote prolonged infection [27]. For example, *Staphylococcus aureus* has previously been shown to transit to autophagosomes shortly after invasion of mammalian cells, and also was unable to replicate in an autophagy-deficient cell line [28].

Although the presence of a TIR domain in BaTdp intuitively suggests a role for this protein in TLR signalling, the findings presented in this paper do not support its involvement in the regulation of the TLR signalling pathway. Rather, we show that BaTdp co-localises with microtubules during expression in a model cell line, HEK. In addition, the expression of BaTdp in HEK cells also increases the levels of lipidated endogenous cytosolic microtubule-associated protein 1A/1B-light chain 3 (LC3), associated with autophagosome formation. Autophagy is known to be an important host-survival strategy [29] and interestingly, *in vivo* experiments in a murine anthrax model revealed that a BaTdp knockout (*ΔBaTdp*) strain was able to colonise host lung tissue significantly faster than WT *B*. *anthracis*, in addition to an increase in lethality. These data indicate that BaTdp differs functionally from previously characterised bacterial Tdps. Rather, BaTdp might be exploited by the host for autophagy-mediated survival during an infection by wildtype *B*. *anthracis*. The more rapid rate of colonisation of the mice to the *ΔBaTdp* strain indicates a plausible lack of autophagic protection due to the absence of BaTdp, and hence greater susceptibility of the mice.

## Materials and Methods

### Animals

All animal studies were approved by the Dstl Animal Welfare and Ethical Review Body, and according to the requirements of the UK Animal (Scientific Procedures) Act 1986. Specific pathogen free 6–8 week old A/J mice were sourced from Charles River laboratories (Margate UK).

### Cell Culture and General Reagents

HEK293T, HEK293 cells stably expressing hTLR2 (HEK-TLR2) and HEK293 cells stably expressing MD2, CD14 and hTLR4 (HEK-TLR4) were purchased from Invivogen and maintained in Dulbecco’s Modified Eagle Medium (DMEM) supplemented with 10% (v/v) fetal bovine serum (FBS) and appropriate antibiotics at 37°C, 5% CO_2_, under humidified environment. Antibodies to GAPDH and β tubulin were from Santa Cruz Biotechnology, LC3B was from Cell Signaling Technology, His and FLAG were from Sigma-Aldrich, rabbit IgG Alexa Fluor 568 conjugate was from Invitrogen. *E*. *coli* 055:B5 lipopolysaccharide (LPS) and lipoteichoic acid (LTA) were from Sigma-Aldrich. Mitotracker dye was from Invitrogen.

### Construction of Plasmids

PCR primers 5′-caccatgtattatcatattagaatta-3′ and 5′-atacgtaacttttaatccagc-3′ were used to amplify the *BaTdp* gene from *B*. *anthracis* genomic DNA. PCR product was then cloned into the mammalian expression vector pcDNA3.2/VS/GW/D-TOPO^®^ (Invitrogen) and transformed into *E*. *coli* TOP10 cells (Invitrogen). For localisation studies, the BaTdp gene was cloned into a pEGFP-C1 vector (Clontech). Bacterial plasmid for expression of the BaTdp TIR domain (BaTIR) tagged with GB1 and His-tag was generated by cloning the sequence corresponding to amino acids D126-R266 of BaTdp, into GEV2 [30]. The four human adaptor protein expression constructs were made in previous studies by individually cloning the TIR domain-encoding region of human MyD88 and TRIF (MyD88-TIR and TRIF-TIR), as well as full length MAL and TRAM into the pGEX-6P-1 plasmid (GE Healthcare), which contains a gene encoding a GST tag [31]. Plasmids coding for G164A mutant BaTdp were generated with a QuikChange site-directed mutagenesis kit (Stratagene) using complementary primers 5′-ggaagctaatgaagcgttaacagttcttg-3′ and 5′-caagaactgttaacgcttcattagcttcc-3′, following the manufacturer’s instructions.

### Expression and Purification of BaTIR and Human Adaptor Protein Constructs

Bacterial protein expression was performed in BL21 (DE3) *E*. *coli* cells (Invitrogen). GB1-BaTIR-His was purified on Co^2+^-IMAC Talon resin (Clontech), while GST-tagged MyD88-TIR, TRIF-TIR, MAL and TRAM were purified on Glutathione Sepharose HP resin (GE Healthcare). Following purification, the removal of imidazole or reduced glutathione was performed by overnight dialysis, followed by concentration to 1–5 mg/mL using Amicon ultrafiltration (10 kDa MWCO) filters. The purity of the protein was assessed by SDS-PAGE analysis and proteins were flash frozen in liquid nitrogen and stored at -80°C until used.

### Determination of Protein Concentration

After purification, dialysis and ultrafiltration, the protein concentration was determined spectrophotometrically at OD 280 nm and calculated using the Beer-Lambert law.

### GST Pull-Down Assays

100 μg GST-tagged bait protein MyD88-TIR, TRIF-TIR, MAL or TRAM was bound to 50 μL glutathione resin pre-equilibrated with binding buffer [20 mM Tris-HCl pH 7.5, 150 mM NaCl, 5 mM DTT, 1 mM EDTA, 0.1% v/v Tergitol^®^ type NP-40] and incubated at 4°C for 1 hour. The resin was washed with 1 mL binding buffer, before 60 μg of GB1-BaTIR-His was added as the prey protein. After an additional 1-hour incubation at 4°C, the resin was washed with binding buffer four times and total protein content was eluted by boiling the resin in 50 μL SDS-PAGE sample buffer at 95°C for 10 minutes. Samples were analysed by Western blot with bands visualised using SIGMAFAST BCIP^®^/NBT (Sigma).

### Confocal Microscopy

HEK-TLR4 cells were grown on coverslips in 12-well plates, transfected with Turbofect reagent (Thermo Scientific) and incubated for an additional 24 hours. Cells were washed with PBS and fixed with 4% paraformaldehyde in PBS for 15 minutes, followed by washing three times with PBS. Cells were permeabilised with 0.1% Triton X-100 and stained with a primary antibody targeting β-tubulin and a secondary fluorescent antibody. Cells were mounted with Prolong Gold Antifade mounting media containing DAPI (Invitrogen) and visualised under a META 510 confocal laser scanning microscope (Zeiss).

### MTT Cell Viability Assay

HEK293T cells were grown in 96-well plates and transfected with 100 ng of plasmid per well using Turbofect reagent (Thermo Scientific). To estimate overall cell viability, 20 μL of 3-(4,5-dimethylthylthiazol-2-yl)-2,5-diphenyltetrazolium bromide (MTT) salt (Sigma) solubilised in PBS at 5 mg/mL was added to each well, followed by further incubation at 37°C for 4 hours. Formazan crystals were then solubilised overnight by adding 100 μL 10% SDS in 10 mM HCl to each well. Metabolic activity of viable cells was then measured at 590 nm with a Synergy Mx microplate reader (BioTek).

### *In Vitro* Cell PAMP Stimulation and Measurement of Secreted Cytokines

HEK-TLR2 or HEK-TLR4 cells were seeded in 24-well plates and transfected with pCDNA plasmids encoding either BaTdp or BaTdp (G164A), using Turbofect reagent (Thermo Scientific). Total plasmid concentration was normalised to 1 μg/mL growth medium using empty pCDNA vector. 24 hours post-transfection, cells were stimulated with either 0.1 μg/mL LPS or 1 μg/mL LTA for 24 hours, after which cell supernatants were harvested and secreted amounts of inflammatory cytokines, IL-8 and TNFα, were quantified with OptEIA human cytokine ELISA kits (BD BioSciences), following manufacturer’s instructions.

### Measurement of Cellular Autophagy

To measure autophagic flux, cellular LC3B was detected by western blot analysis. HEK293T cells were seeded in 6-well plates and transfected with plasmids encoding either BaTdp or BaTdp (G164A) using Turbofect reagent. Total plasmid concentration was normalised to 1 μg/mL growth medium using empty pCDNA vector. At 24 and 48 hours post-transfection, cells were harvested and lysed by boiling in PBS supplemented with 1% SDS. Lysates were resolved by SDS-PAGE and analysed for LC3B content by Western blot with bands visualised using enhanced chemiluminescent (ECL) HRP substrate (Thermo Scientific) and detected with an ImageQuant LAS 4000 mini system (GE Healthcare).

### Construction of a *B*. *anthracis ΔBaTdp* Strain

A deletion construct of the BaTdp gene suitable for allelic exchange was created by cloning two NotI-XmaI DNA fragments containing BaTdp flanking regions into the NotI site of pBKJ236 [32]. These fragments were created by PCR with primers 5′-GGATCCgatcgatggcatacgtcata-3′ and 5′-CCCGGGcataatatccctcgactttc-3′ upstream and 5′-CCCGGGtaaaatcgcgaactgtttgc-3′ and 5′-GCGGCCGCaacggtttcaactcctac-3′ downstream of the BaTdp gene (capitalised bases denote BamHI, XmaI and NotI sites). Ligation of the two fragments created a precise deletion of the BaTdp open reading frame from the start to the stop codon, inclusive, replacing it with an XmaI site. The resulting construct was used to perform allelic exchange in *B*. *anthracis* STI to create a BaTdp null mutant by a procedure described previously [32]. In brief, integrants of the BaTdp-pBKJ236 plasmid construct were isolated by a shift to the replication-nonpermissive temperature after conjugative transfer and growth at the permissive temperature while maintaining selection for erythromycin resistance. A second plasmid, pBKJ223, was then introduced by conjugation and selection for tetracycline resistance. This plasmid mediates cleavage within the vector sequences, thus stimulating recombination and the loss of the integrated plasmid, resulting in gene replacement in a portion of the erythromycin-sensitive candidates. The absence of the BaTdp locus in *B*. *anthracis* STI Δ*BaTdp* was demonstrated by PCR using primers 5′-gatccggacataatggatgc-3′ (upstream) and 5′-tcagccttaccttctccttc-3′ (downstream). These primers were designed to bind to sequences flanking the region included in the deletion construct described above. The *B*. *anthracis* STI Δ*BaTdp* construct was also tested by PCR to ensure the retention of pX01 using primers to *pagA;* 5′-agtgcatgcgtcgttctttgata-3′ (upstream) and 5′-gaatttgcggtaacacttcactcc-3′ (downstream).

### *In Vivo* Studies

To determine the median lethal dose (MLD) of *B*. *anthracis* STI Δ*BaTdp*, ten groups of 6 female 6–8 week old A/J mice were infected with a range of doses (10^3^–10^6^ spores) of *B*. *anthracis* STI or *B*. *anthracis* STI Δ*BaTdp* via the intranasal route. Mice were monitored for 10 days before surviving mice were culled. MLD were calculated using the Reed and Muench method [33]. To determine differences in bacterial colonisation, eight groups of 6 female 6–8 week old A/J mice were infected via the intra-nasal route with 1.75×10^3^ spores of *B*. *anthracis* Δ*BaTdp* or 1.9×10^3^ spores *B*. *anthracis* STI. Lungs, spleen and kidney were harvested from mice at 3, 4, 5 or 6 days post-challenge for bacteriological analysis. Whole lung tissue was homogenized by passing through a 70 μm cell strainer (BD Biosciences) in 1 mL PBS and 10-fold duplications were plated onto L-agar plates for enumeration, calculated as CFU/mL.

### Statistical Analysis

*In vitro* data are presented as means ± SD of at least three independent experiments. Comparison with controls were made using a two-tailed Student’s t-test performed in GraphPad Prism^®^ 5, with P values <0.05 considered significant. Cox regression was performed using SPSS V21.0, where strain was a factor and log_10_ dose a covariate. *In vivo* bacterial burden data was transformed to the log_10_ and then analysed using a two parameter General Linear Model using SPSS V21.0. Bacterial burden data from dead mice was not included in the analysis.

## Results

### *Bacillus anthraci*s Tdp Is Phylogenetically Distinct from Mammalian TIR Domain-Containing Proteins

**Fig 1:**
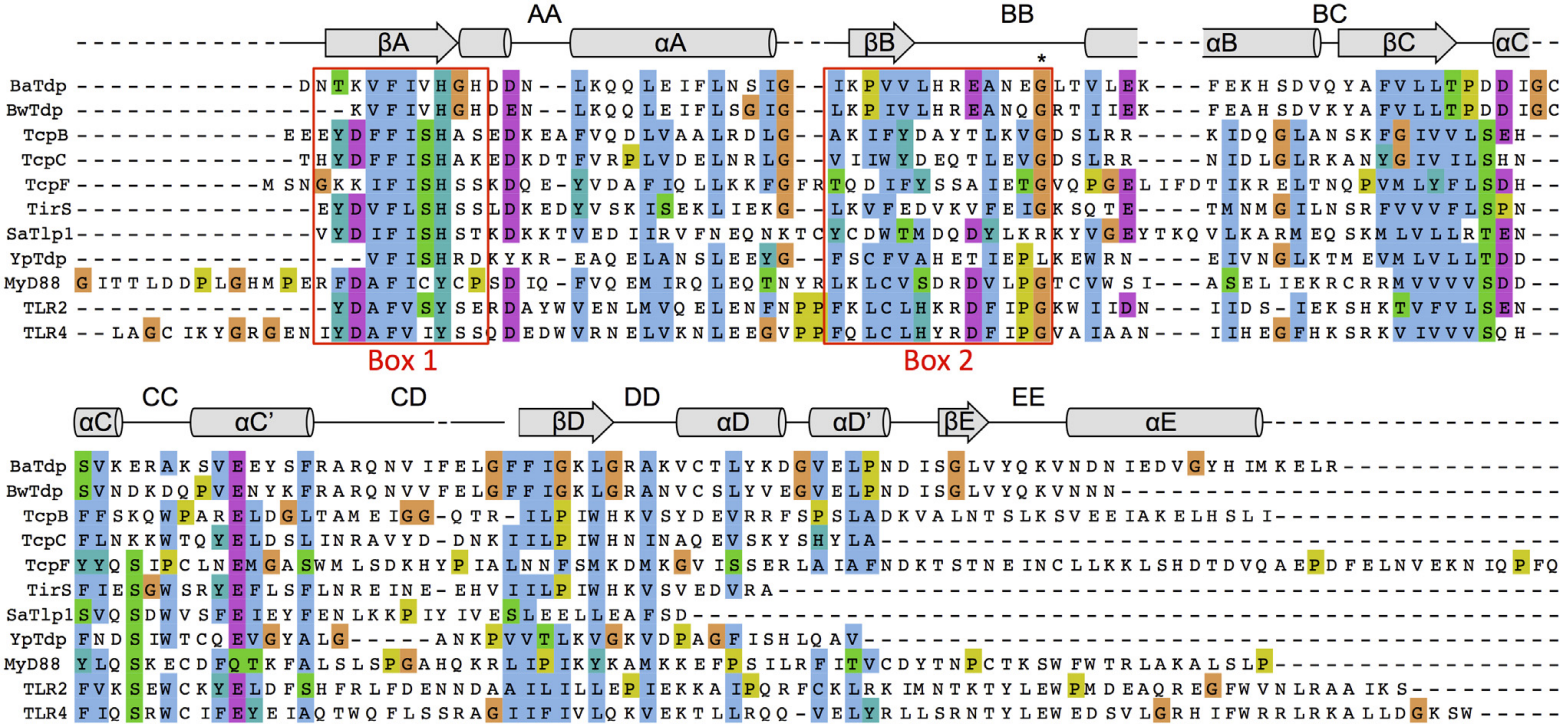
Sequence alignment of TIR domain proteins. Sequence alignment of the TIR domains from BaTdp (BA_4098, residues 126–266), BwTdp from *Bacillus weihenstephanensis* (KEZ82977.1, residues 132–256), TcpB from *Brucella melitensis* (NP5404591.1, residues 116–250), TcpC from *Escherichia coli* CFT073 (NP_754290.1, residues 169–280), TcpF from *Enterococcus faecalis* (CCO72761.1, residues 3–160), TirS from *Staphylococcus aureus* (WP_000114516.1, residues 142–246), SaTlp1 from *S*. *aureus* (CAQ50581.1, residues 202–308), YpTdp from *Yersinia pestis* (NP_ 669733.1, residues 139–240), and human proteins MyD88 (AAH13589.1, residues 146–296), TLR2 (AAH33756.1, residues 641–784) and TLR4 (NP_003257.1, residues 621–781). The Box 1 and Box 2 regions are indicated by red boxes. The conserved glycine residue in the box 2 region is marked with an asterisk. Structural features indicated are based on the TcpB structure [19]. The alignment was generated using MAFFT v.7220 [35] and visualised in Jalview [36].

Our previous bioinformatics survey [10] identified one protein that contains a TIR domain in *B*. *anthracis* strains (BaTdp). This protein is conserved across *B*. *anthracis* strains and displays substantial homology (66% identity) to a predicted Tdp found in the *Bacillus weihenstephanensis* genome (Fig 1). The genomic sequence context of BaTdp suggests it is in a region of phage DNA. To confirm the evolutionary resemblance of BaTdp to TIR domain proteins, a reverse PSI-BLAST (with BaTdp as the seed) was performed. This analysis highlighted a TIR domain protein from *Dictyostelium discoideum* AX4 and one from *Ricinus communis*, suggesting that BaTdp is phylogenetically unrelated to mammalian TIR domain proteins. Although the BaTdp sequence contains the Box 1 and Box 2 regions characteristic of the TIR domains, the 3D structure prediction tool, FUGUE [34], did not find any highly scoring matches (data not shown). This suggests that at the level of its primary sequence, part of the BaTdp is homologous to TIR domains but the protein may differ at a tertiary level from previously solved structures of TIR domain proteins. Furthermore, the functionally important BB loop in the BaTdp lacks the Pro residue usually found in mammalian TLR-TIRs and adaptor TIRs as well as some bacterial Tdps, such as YpTdp. However, the BaTdp BB loop does contain the conserved glycine residue, Gly164 (Fig 1). The corresponding Gly158 residue in the *B*. *melitensis* TcpB protein has been shown to be important for the protein’s ability to suppress TLR2-mediated NFκB activation [16], and is well conserved across both mammalian and bacterial Tdps.

### The TIR Domain of BaTdp Interacts with a Range of Mammalian Adaptor Proteins *In Vitro*

**Fig 2:**
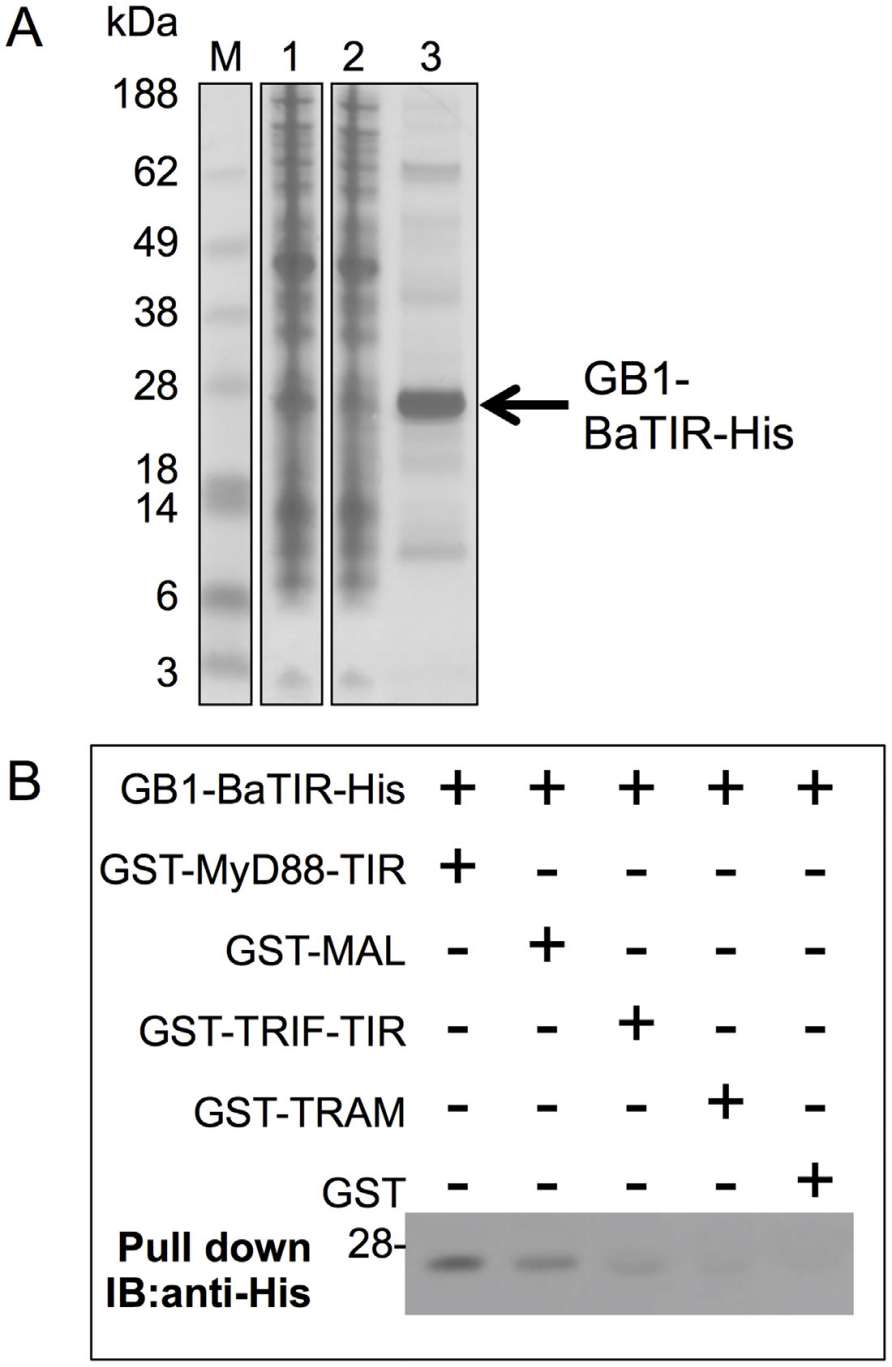
The TIR domain of BaTdp interacts with a range of mammalian adaptor proteins *in vitro*. (A) SDS-PAGE analysis of the GB1-BaTIR-His construct following isolation using Co^2+^ affinity chromatography. M, molecular weight markers; lane 1, soluble fraction of whole cell lysate; lane 2, flowthrough during purification; lane 3, purified protein. The band corresponding to GB1-BaTIR is indicated by the arrow. Figure shows cropped lanes from non-adjacent lanes of the same gel. (B) Western blot analysis of samples obtained from GST pull down interaction assays. IB, immunoblot. Result shown is representative of at least three independent experiments. Image has been cropped to display only the region of interest. An uncropped version of the blot is shown in S1 Fig.

The recombinant expression of full length bacterial Tdps is notoriously challenging [14, 37], so in order to explore whether BaTdp could interact with mammalian adaptor proteins, we focused on the isolated TIR domain (BaTIR, residues D126-R266). This approach has allowed us to successfully study the interactions of the TIR domain protein from *Yersinia pestis* [37]. BaTIR was expressed in *E*. *coli* BL21 cells as a fusion protein with an N-terminal GB1 tag and a C-terminal hexahistidine tag using the GEV2 vector. The protein expressed well and could be readily isolated using Co^2+^ affinity chromatography with a final yield of 1.25 mg/L LB culture. SDS-PAGE analysis revealed that the sample contained one dominant species appearing just below the 28 kDa marker band (Fig 2A). The GB1 tag is approximately 6 kDa in size with BaTIR having a theoretical molecular weight of 16 kDa. The final concentration of the sample was approximately 5 mg/mL following dialysis and ultrafiltration.

Pull down experiments were performed using GST tagged versions of MyD88-TIR, TRIF-TIR, MAL and TRAM as baits, with the isolated GB1-BaTIR-His as prey. Interestingly, GB1-BaTIR-His interacted with MyD88-TIR (Fig 2B), with possible weak interactions also detected using MAL and TRIF-TIR. No interaction was detected between GB1-BaTIR-His and GST only, confirming the specificity of the interaction (Fig 2B). MyD88 is the key TLR adaptor used by all human TLRs except TLR3 for downstream immune activation, and thus the results indicated that BaTdp may have a role in modulating host TLR signalling. We therefore further investigated this potential function of BaTdp in a mammalian cell environment.

### BaTdp Co-Localises with Tubulin in HEK-TLR4 Cells and Is Not Cytotoxic

**Fig 3:**
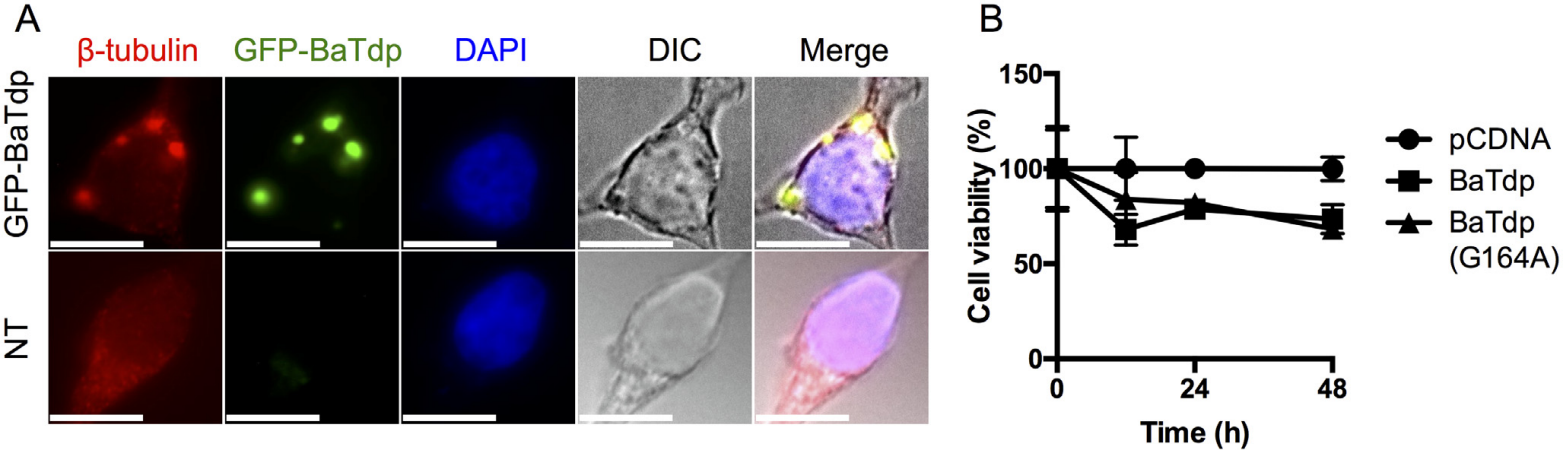
BaTdp co-localises with cellular tubulin. (A) HEK-TLR4 cells transfected with plasmid coding for BaTdp-GFP fusion protein were fixed, permeabilised and stained with a β-tubulin antibody and visualised with a red fluorescent secondary antibody. The protein appeared in the cytosol mainly as condensed spots. Cell nuclei were stained with 4',6-diamidino-2-phenylindole (DAPI). All scale bars, 10 μm. Original magnification ×100. DIC, Differential interference contrast. (B) Overall cell viability of HEK293T cells transfected with plasmid encoding BaTdp or BaTdp (G164A) was estimated by MTT assay. Plasmid amount was normalised to 100 ng per well (corresponding to 1000 ng/mL) using empty pCDNA control plasmid. Bars represent mean values of three independent experiments. *Error bars*, SD of triplicates.

In order to explore the functional role of BaTdp in a cellular environment, we expressed the protein in HEK-TLR4 cells, a model cell line used regularly to assess TLR4-signalling initiated by LPS stimulation [6]. Initial studies focused on the localisation of a GFP tagged version of the protein. The BaTdp-GFP protein localised into discrete cytosolic structures mainly near the cell membrane, and due to the patterns formed, we speculated that the protein co-localises with tubulin, as seen for TcpB from *B*. *melitensis* [16]. BaTdp-GFP was seen in the cells mainly as distinct spots (Fig 3A), and cellular staining with an antibody targeting β-tubulin, followed by fluorescent staining, revealed a clear overlap between the two proteins, suggesting that BaTdp targets microtubule networks in the cell. It is not clear whether the co-localisation seen is due to a direct interaction between BaTdp and tubulin. We did not observe any noticeable shrinkage or rounding of cells expressing BaTdp, which has been reported during TcpB expression in HEK cells [16]. To assess whether the protein had a cytotoxic effect on HEK cells, an MTT assay was used to determine the overall cellular viability up to 48 h post-transfection. A cell line derived from HEK293 containing the SV40 T-antigen (HEK293T) was used to ensure a high level of protein expression, and as shown in Fig 3B, expression of BaTdp (both WT and G164A mutant protein) was comparable and associated with only minor reduction in cell viability, confirming that BaTdp does not elicit a cytotoxic effect on the host cell.

### BaTdp Does Not Affect TLR Signalling in Mammalian Cells

**Fig 4:**
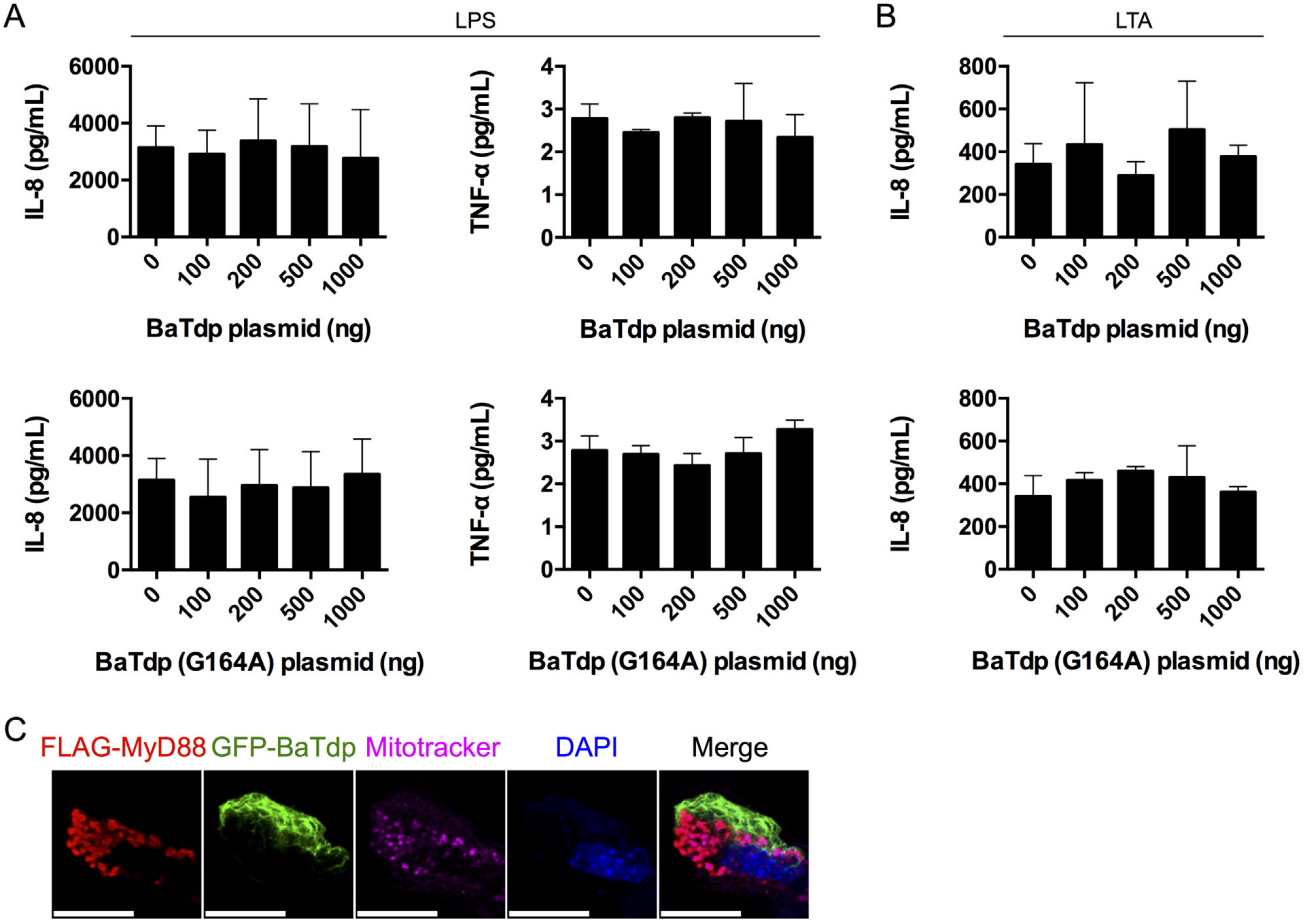
*In vitro* signalling assays indicate that BaTdp does not affect TLR signalling. (A) HEK-TLR4 cells were transfected with increasing amounts of vector DNA containing the gene encoding BaTdp (wildtype) or the BaTdp (G164A). 24 hours post-transfection, cells were challenged with LPS (0.1 μg/mL) and the production of inflammatory cytokines, TNFα and IL-8, was assessed by ELISA 24 hours later. Bars represent mean values of three independent experiments. *Error bars*, SD of triplicates. (B) HEK-TLR2 cells were transfected with plasmid encoding for BaTdp or BaTdp (G164A) and challenged with LTA (1 μg/mL) 24 hours post-transfection, followed by assessment of IL-8 content in the culture supernatant by ELISA after an additional 24 hours. Bars represent mean values of three independent experiments. *Error bars*, SD of triplicates. (C) HEK-TLR4 cells were co-transfected with plasmids encoding FLAG-tagged MyD88 and GFP-tagged BaTdp. 24 hours post transfection, after being stained with mitotracker dye to stain cellular mitochondria, cells were fixed, permeabilised and stained with an anti-FLAG antibody and visualised with a red fluorescent secondary antibody. All scale bars, 10 μm. Original magnification ×100.

As shown above, our GST pull-down results indicated that the TIR domain of BaTdp, BaTIR, specifically interacts with human TLR adaptor proteins, which prompted us to speculate that the expression of BaTdp in mammalian cells may disrupt TLR-mediated downstream signalling, as previously described for multiple bacterial Tdps. HEK-TLR4 cells were initially transfected with plasmid coding for BaTdp and incubated for 24 h, followed by stimulation with 0.1 μg/mL LPS for an additional 24 h. Contrary to expectation, the expression of BaTdp had no significant effect on LPS-induced production of either TNFα or IL-8 at any of the concentrations of vector DNA transfected (0–1000 ng; Fig 4A), indicating that BaTdp does not have a role in TLR4 signalling. It is possible that this is due to the fact that *B*. *anthracis* is a Gram-positive bacterium and does not possess LPS as a stimulant for TLR4. However, TLR4 is the receptor for a wide variety of molecules in addition to LPS, including anthrolysin O [38] which is known to be expressed by *B*. *anthracis* [39], thus LPS should be an appropriate ligand to use to assess the effects of BaTdp on TLR signalling. Nevertheless, we decided to further conduct a similar experiment in HEK cells stably expressing TLR2, whose natural ligands include LTA from the cell wall of Gram-positive bacteria. BaTdp expression in HEK-TLR2 cells was again found to have no significant effect on IL-8 secretion following LTA-stimulation (Fig 4B), suggesting that unlike many other bacterial TIR domain proteins, the primary functional role of BaTdp is not to suppress TLR-mediated immune activation. The levels of TNFα secretion from HEK-TLR2 cells were also assessed following LTA stimulation, but were found to be below the assay detection limit (data not shown). In addition to the cytokine secretion assays shown here, cell reporter assays were performed to assess if BaTdp-expression had any effect on IL-1β-, TNF- or LPS-induced NFκB activation in HEK cells, but no significant differences could be detected (data not shown). In each experiment, the amount of TNFα secreted from cells following PAMP-stimulation was consistently low and near the assay detection limit, making this data of limited use. In contrast, PAMP-stimulation of cells resulted in IL-8 secretion levels markedly higher than unstimulated control cells. To ensure the fidelity of the assays, control experiments were performed with cells expressing a known inhibitor of TLR-signalling (S2 Fig).

HEK-cells were also co-transfected with plasmids encoding GFP-tagged BaTdp and FLAG-tagged MyD88, followed by staining with anti-FLAG antibody and analysis by fluorescence microscopy. As shown in Fig 4C, MyD88 localised to mitochondria as previously described [31], but no noticeable overlap between MyD88 and BaTdp could be observed, indicating that BaTdp does not target MyD88 in a cellular environment.

### BaTdp Expression Induces Autophagy in Mammalian Cells

**Fig 5:**
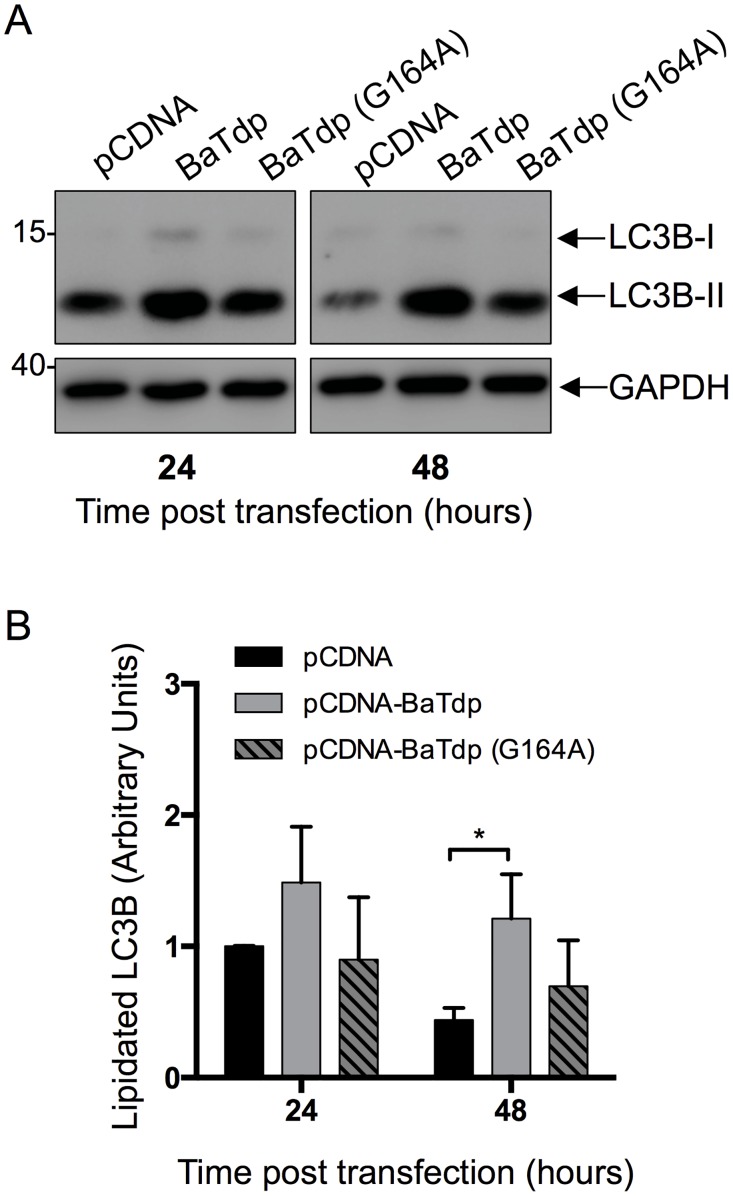
Expression of BaTdp increases lipidation of cellular LC3. (A) HEK293T cells were transfected with plasmid encoding either BaTdp (wild type) or BaTdp (G164A) protein. After 24 or 48 hours post-transfection, cells were lysed in PBS containing 1% SDS and lysates were resolved by SDS-PAGE and analysed by Western blot using an anti-LC3B primary antibody or anti-GAPDH antibody for loading control. Result shown is representative of three independent experiments. Figures show cropped lanes from non-adjacent lanes of the same blot. Images have been cropped to only show the regions of interest. (Uncropped versions of blots are shown in S1 Fig) (B) Western blots were analysed by densitometry using ImageJ software [43]. For each sample, the LC3B-II value was normalised against corresponding GAPDH value. Bars represent mean values of three independent experiments. *Error bars*, SD of triplicates. *P < 0.05 (by two-tailed Student’s t-test).

The co-localisation of BaTdp to tubulin (Fig 3), which is known to play an important role in autophagosome formation in mammalian cells [40], prompted us to hypothesise that BaTdp might be involved in the host cell autophagy. We used the lipidation of LC3-I to LC3-II (specifically in LC3B, which is one of multiple LC3 isoforms) in HEK293T cells as a marker for autophagy activity and followed the process by immunoblotting of cell lysates. This is an established method for monitoring autophagy and autophagy-related processes previously used with HEK cells [41, 42]. To ensure the complete extraction of lipidated LC3B during cell lysis, harvested cells were boiled in PBS containing 1% SDS before analysis by SDS-PAGE and subsequent Western blot. WT BaTdp expression was found to be associated with significantly increased levels of LC3B-II at 48 h post-transfection (Fig 5), while BaTdp (G164A) expression did not result in any significant difference in cellular LC3B-II content compared with cells transfected with empty plasmid. This suggests that Gly164, located in the conserved Box 2 region of the TIR domain, is functionally associated with LC3B-mediated autophagy.

**Fig 6:**
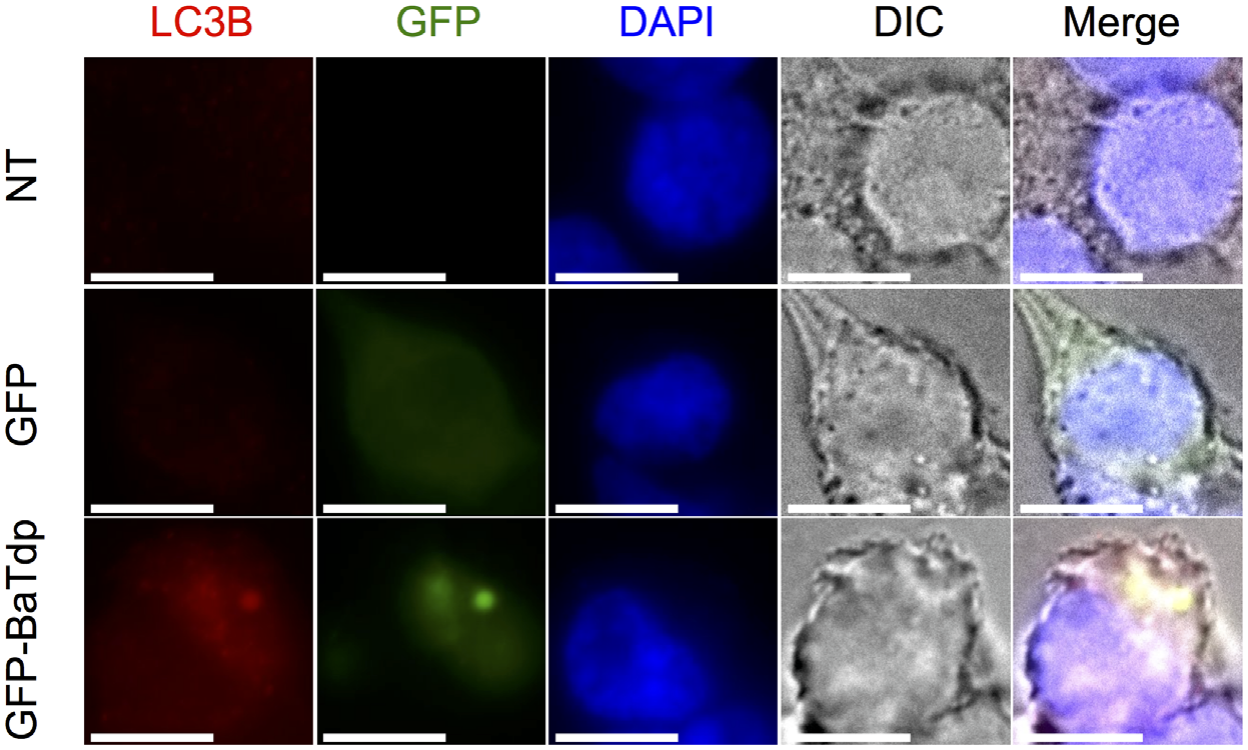
BaTdp co-localises with cellular LC3. HEK293T cells were transfected with plasmid encoding GFP-tagged BaTdp. 24 hours post transfection, cells were fixed, permeabilised and stained with an anti-LC3B antibody and a secondary red fluorescent antibody, followed by analysis by fluorescence microscopy. Non-transfected cells, or cells transfected plasmid encoding GFP were used as control. All scale bars, 10 μm. Original magnification ×100.

HEK-cells expressing GFP-tagged BaTdp were also stained with anti-LC3B antibody, followed by analysis by fluorescence microscopy. As shown in Fig 6, BaTdp expression was associated with clear co-localisation and accumulation of LC3B, further suggesting that BaTdp interacts with cellular autophagosomes.

### *B*. *anthracis* STI Δ*BaTdp* Virulence

Given that BaTdp had been found to localise to the cellular microtubule structures and induce autophagosome formation, but did not appear to act on intracellular TLR signalling, we were curious to see if a *B*. *anthracis ΔBaTdp* strain would display a reduction in lethality. To test this, mice were infected with a range of doses of either wild type *B*. *anthracis* STI or *B*. *anthracis* STI Δ*BaTdp* and then monitored for 10 days before being culled. The Reed and Muench calculation [33] was then used to calculate the MLD of *B*. *anthracis* STI Δ*BaTdp*. The MLD of *B*. *anthracis* STI Δ*BaTdp* was calculated to be 5.25×10^3^ spores per mouse, higher than the previously determined value of 1.34×10^3^ for WT *B*. *anthracis* STI [44], but still within the margin of error. This data was further analysed using Cox regression and we found the likelihood of survival time effect was low (P = 0.418).

**Fig 7:**
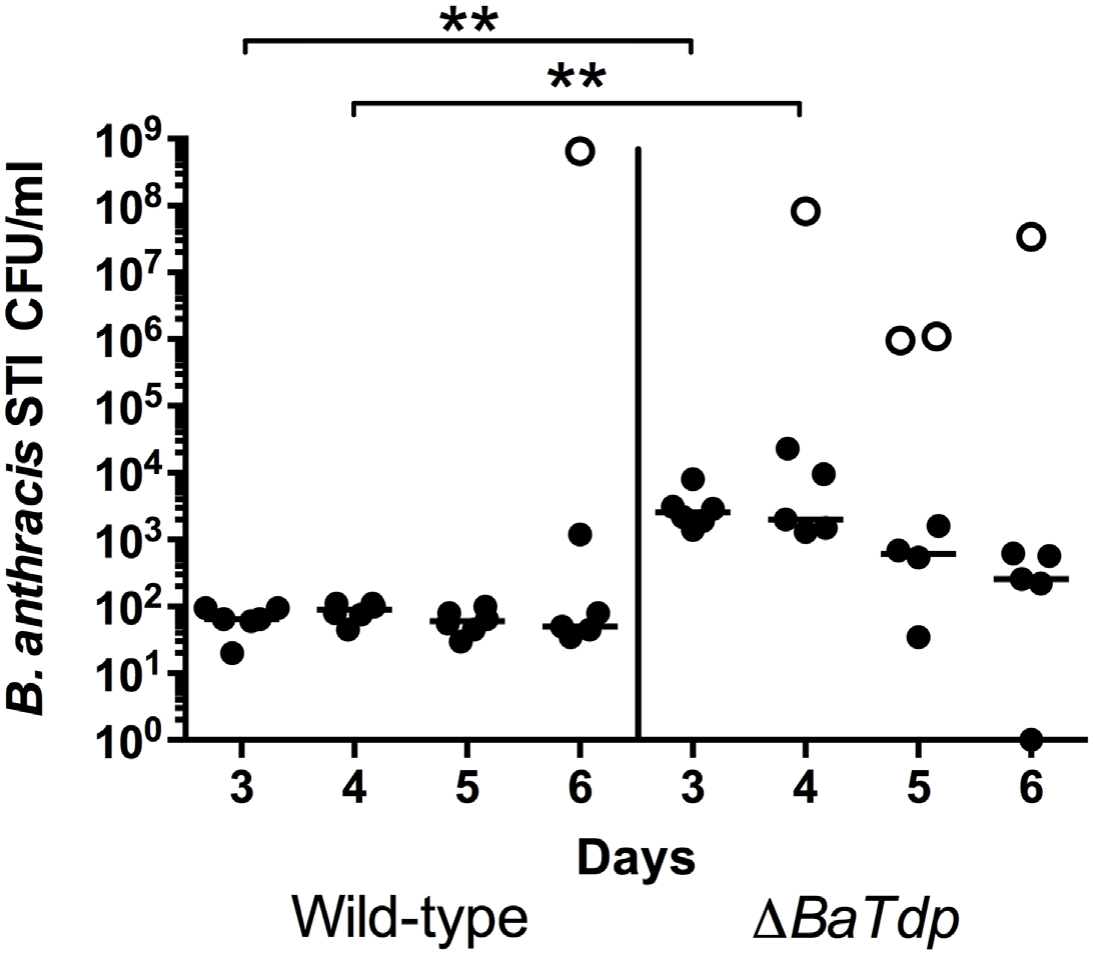
Colonisation of lungs by *B*. *anthracis* STI or *B*. *anthracis* STI Δ*BaTdp* during murine model of infection. Eight groups of 6 6–8 week old A/J female mice were infected via the intra-nasal route with either *B*. *anthracis* STI or *B*. *anthracis* STI Δ*BaTdp*. At 3, 4, 5 or 6 days post-infection, lungs were removed from mice and bacterial numbers enumerated. Open symbols indicate mice that died during the experiment. The bar represents the median bacterial load for each group. WT = Wild Type *B*. *anthracis* STI, MT = Mutant *B*. *anthracis* STI Δ*BaTdp*. Data points show the bacterial burden per lung. Significance markers indicate the output of Bonferroni’s post-tests where P < 0.01. Bacterial burden data from dead mice was not included in the analysis.

In a separate experiment, A/J mice infected with either wild type *B*. *anthracis* STI or *B*. *anthracis* STI Δ*BaTdp* were culled at 3, 4, 5 and 6 days. Their spleens, lungs and kidneys were removed for analysis to discern any differences between their bacterial loads. Data indicated that no viable bacteria were isolated from the spleen or kidney unless the animal had died, with the exception of two mice infected with wild type *B*. *anthracis* at day 5 and a single mouse at day 3 infected with *B*. *anthracis* STI Δ*BaTdp* (data not shown). Lungs were colonised by *B*. *anthracis* STI Δ*BaTdp* to a greater extent than the wild type strain at day 3 (P < 0.004) and day 4 (P = 0.008) but there was no statistically significant difference at the later time points (Fig 7). Together these data indicate increased virulence of *B*. *anthracis* as a result of deletion of *BaTdp*.

## Discussion

This paper details an investigation into the TIR-containing protein from the highly pathogenic bacterial species, *B*. *anthracis*, and its overall effect on virulence. *B*. *anthracis* is a tier 1 pathogen classified by the United States Centers for Disease Control and Prevention (CDC), due to its potential as a bioterrorism agent. The need for development of effective therapeutic treatments against *B*. *anthracis* infection was further highlighted in 2001 when anthrax toxin was used during a series of attacks in the United States, but functional studies are by nature associated with strict technical difficulties.

Proteins containing TIR domains have been discovered through *in silico* methods in a wide variety of bacterial species. Early studies revealed that these proteins play a role in the subversion of a host immune response through negative regulation of the TLR signalling pathway. However, this unified theory may be too simplistic, as other possible functions of these proteins are likely, particularly as many TIR domain proteins are present in non-pathogenic bacteria [10].

The majority of bacterial Tdps characterised to date have been from Gram-negative species, although recent studies describe Tdps from Gram-positive species. Askarian and colleagues reported the characterisation of an *S*. *aureus* Tdp, TirS, that appears to block TLR2-mediated NFκB activation *in vitro* [45], although mice infected with WT or KO strains displayed comparable survival rates. An *Enterococcus faecalis* Tdp, TcpF, has also been shown to suppress TLR2-dependent NF*κ*B activation in cultured eukaryotic cells [46]. Patterson and colleagues later identified and characterised two proteins, SaTlp1 and SaTlp2, from *S*. *aureus* harbouring TIR-like domains [47]. In contrast to previously characterised bacterial Tdps, these two proteins were associated with an upregulation of host immune signalling through NFκB activation [47]. Here, we have characterised the Tdp of a significant tier 1 bioterrorism pathogen. We found that although the BaTdp protein seemed to consistently show no subversive role from the host TLR immune activation *in vitro*, it may be exploited by the host for autophagy-mediated survival during *B*. *anthracis* infection.

Observing an interaction between the BaTdp TIR domain and the human TLR adaptors *in vitro* using GST pull-down, without a discernable effect on TLR signalling in the cell-based assay was intriguing. Pull-down assays did not provide any quantitative information regarding the affinity of interactions, and thus caution needs to be exercised in extrapolating the function of the protein from *in vitro* interaction analysis. For example, previous research indicated interactions between the *B*. *melitensis* TIR domain protein, TcpB and MyD88, TLR4 and MAL, but only the interaction between TLR4 and MAL was inhibited by TcpB [15]. Furthermore pull down experiments using only the TIR domain indicate it is possible that the full length proteins may have folded in a manner that blocks heterotypic TIR-TIR interactions. The complex cellular environment may also contain unknown factors inhibiting BaTdp-mediated protein-protein interactions, which can then only be observed in a minimal artificial buffer system. These factors may explain why we were unable to see any interaction between MyD88 and full-length BaTdp during overexpression in mammalian cells.

There is currently no high resolution structure of BaTdp, thus it is possible that the region predicted to be the TIR domain actually adopts a significantly different fold. This would explain why this protein doesn’t act in quite the same way as other bacterial Tdps. However this seems unlikely, given that structure is typically more conserved than sequence, and that we observe specific interactions with other known TIR-domains. As TIR-domains are widespread among both pathogenic and non-pathogenic bacteria, fungi, archaea and viruses [10], it would seem unlikely that they serve a single consistent function throughout different organisms. Further studies are required to both fully determine the precise function and the mechanism of action of BaTdp and to explore the role of BaTdp in a biological context.

Although *in vitro* TIR-dependent interaction assays carried out here, and expression data in the literature, suggested that BaTdp may have a role in immune evasion, the *in vivo* studies using the A/J mouse model do not provide any evidence to support this. On the contrary, evaluation of colonisation characteristics of wild-type *B*. *anthracis* STI and the Δ*BaTdp* mutant of this strain suggested that the presence of BaTdp inhibits bacterial propagation following infection. The intra-nasal route used for infection is a natural and appropriate route of infection for *B*. *anthracis* spores that mimic real life anthrax infection well, and any major functional difference between WT and Δ*BaTdp* strains should have been detected by characterisation of host tissue following infection. Although differences in tissue colonisation were seen, the results do not indicate that BaTdp is a major virulence factor. Further studies are required to confirm its functional role.

Of particular interest was our observation that BaTdp co-localised with tubulin, indicating that it targets cytoskeletal networks when overexpressed in mammalian cells. Supporting our finding is a report on the observation of a similar pattern of localisation described for the *Brucella*-encoded Tdp, TcpB [16], although unlike BaTdp, the expression of TcpB was also associated with a clear anti-inflammatory effect on the host cells. However, BaTdp expression in cultured HEK293T cells corresponded to significantly increased level of LC3B lipidation, a process associated with autophagosome formation. As lipidated LC3 is known to directly associate with autophagosomes [41] it is an ideal marker for measuring autophagy. Although intra-autophagosomal lipidated LC3 may be degraded by hydrolases during autophagosome fusion with lysosomes, which is why protein inhibitors are typically included in the assay, we were able to see a clear and consistent BaTdp-associated increase of LC3B throughout experiments. Tubulin-associated BaTdp often appeared in the cytoplasm as condensed spots, possibly an indication of protein aggregation, which may in part explain the in LC3B as a general stress-response to these aggregates. Autophagy is a host defence mechanism, in part used to degrade intracellular pathogens, in order to promote host survival following infection [48], but some pathogenic bacteria such as *Anaplasma phagocytophilum* [49], *Coxiella burnetii* [50], and *S*. *aureus* [28], are believed to exploit this mechanism in order to establish a protective niche environment where the pathogen can develop. As no studies have thus far indicated that *B*. *anthracis* utilises autophagosomes during infection, our finding suggests that the host might recruit BaTdp to trigger autophagy for its survival advantage. *Brucella* encoded TcpB has been shown to partly act by stabilising microtubule structures in the host cell [51], an effect that was recently reported to be dependent on a conserved WxxxE motif located adjacent to the αC helix [52]. Although BaTdp lacks the tryptophan residue of this motif, we were still able to observe a clear co-localisation with tubulin in HEK293T cells, suggesting that this is not an isolated occurrence but rather a conserved function that may be shared with other bacterial Tdps. It is not uncommon for intracellular pathogens to target cytoskeletal structures by releasing effector proteins that modulate microtubule dynamics [53], but the significance of doing so, or the mechanisms involved, are not well understood.

Multiple bacterial Tdps have been shown to cross the eukaryotic plasma membrane [11, 54], but *B*. *anthracis* infects a host as spores that germinate intracellularly following engulfment by macrophages. Thus, BaTdp may not necessarily require this ability in order to gain access to the cytosolic environment. The secretion of bacterial Tdps from both Gram-negative and -positive species has previously been described. Cirl and colleagues have demonstrated that TcpC is secreted by *E*. *coli* CFT073 grown in media, followed by internalisation by mammalian cells using a transwell system [11], and TirS has also been described as being released by *S*. *aureus* using similar methodology [45]. However, it remains unclear whether BaTdp is actively secreted during infection.

In conclusion, although the *B*. *anthracis* protein, BaTdp, displays an ability to interact with human Tdps *in vitro* via its TIR domain, it does not appear to play a role in immune evasion. In contrast, the absence of BaTdp in *B*. *anthracis* led to faster lung colonisation following infection. It is possible that the host exploits BaTdp to induce autophagy in order to promote the host’s own survival, but more research is needed to further verify the pathophysiological role.

## Supporting Information

S1 Fig Uncropped versions of Western blots presented. Uncropped versions of Western blots shown in Figs 2 (left) and 5 (middle and right).
(TIFF)

S2 Fig PAMP-induced cytokine activation of HEK-TLR2 and -TLR4 cells. (A) HEK-TLR4 cells were transfected with 1000 ng of empty vector (EV) or a vector containing the gene encoding SARM, a known inhibitor of TLR-signalling. 24 hours post-transfection, cells were challenged with LPS (0.1 μg/mL) and the production of inflammatory cytokines, TNFα and IL-8, were assessed by ELISA 24 hours later. Background levels of cytokines produced by non-transfected (NT) cells without LPS-stimulation were also assessed. Bars represent mean values of three independent experiments. *Error bars*, SD of triplicates. ND, not detected. (B) HEK-TLR2 cells were transfected with 1000 ng of empty vector (EV), followed by assessment of IL-8 content in the culture supernatant by ELISA after an additional 24 hours. Background levels of IL-8 produced by non-transfected (NT) cells without LTA-stimulation were also assessed. Bars represent mean values of three independent experiments. *Error bars*, SD of triplicates. ND, not detected.
(TIFF)
