# Supplementary figures and images for: Bacillus anthracis TIR Domain-Containing Protein Localises to Cellular Microtubule Structures and Induces Autophagy
Source: PLoS One. 2016 Jul 8;11(7):e0158575. doi: 10.1371/journal.pone.0158575 (PMC4938393; doi:10.1371/journal.pone.0158575)

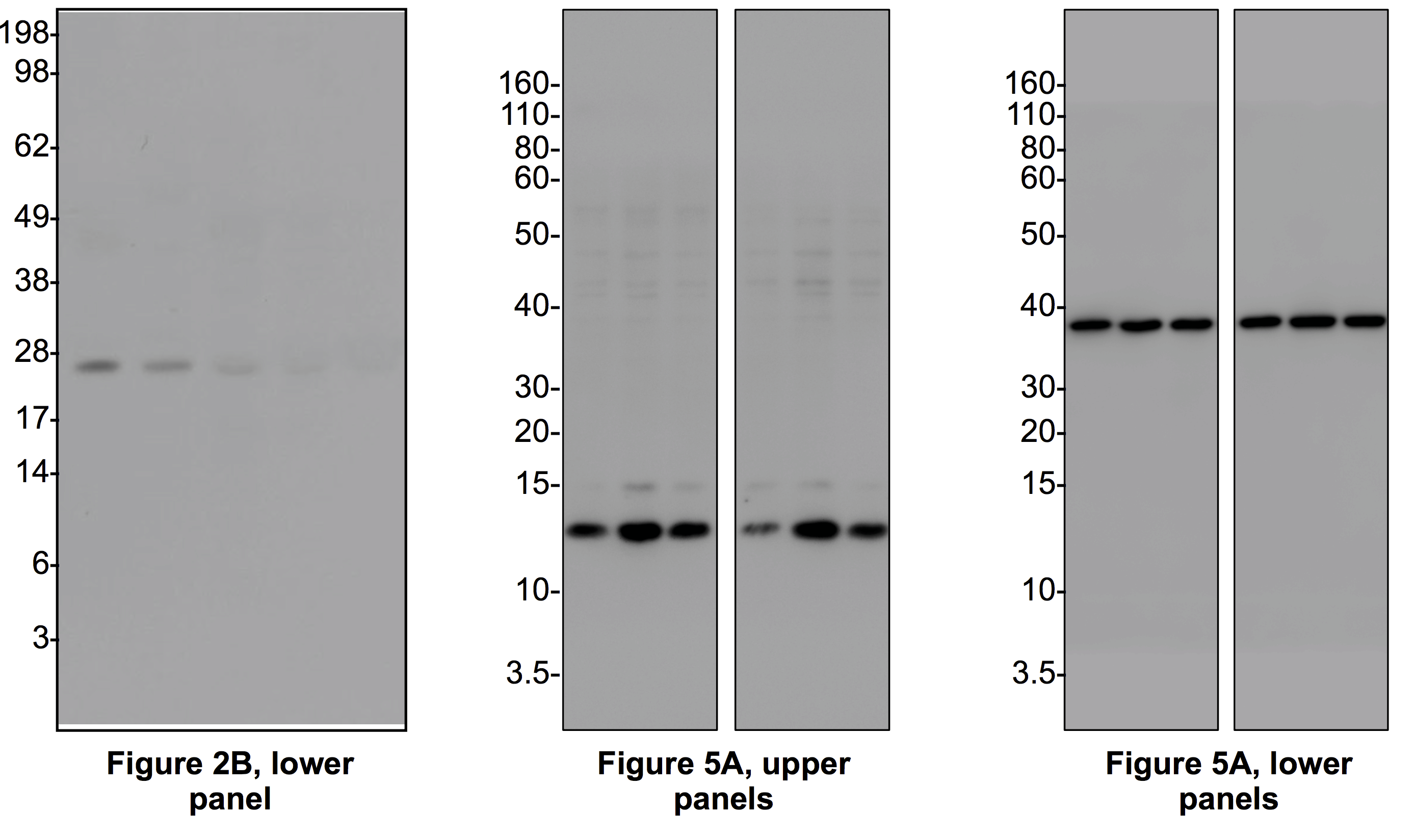

Supplement: S1 Fig — Uncropped versions of Western blots shown in Figs 2 (left) and 5 (middle and right). (TIFF) [file pone.0158575.s001.tiff]

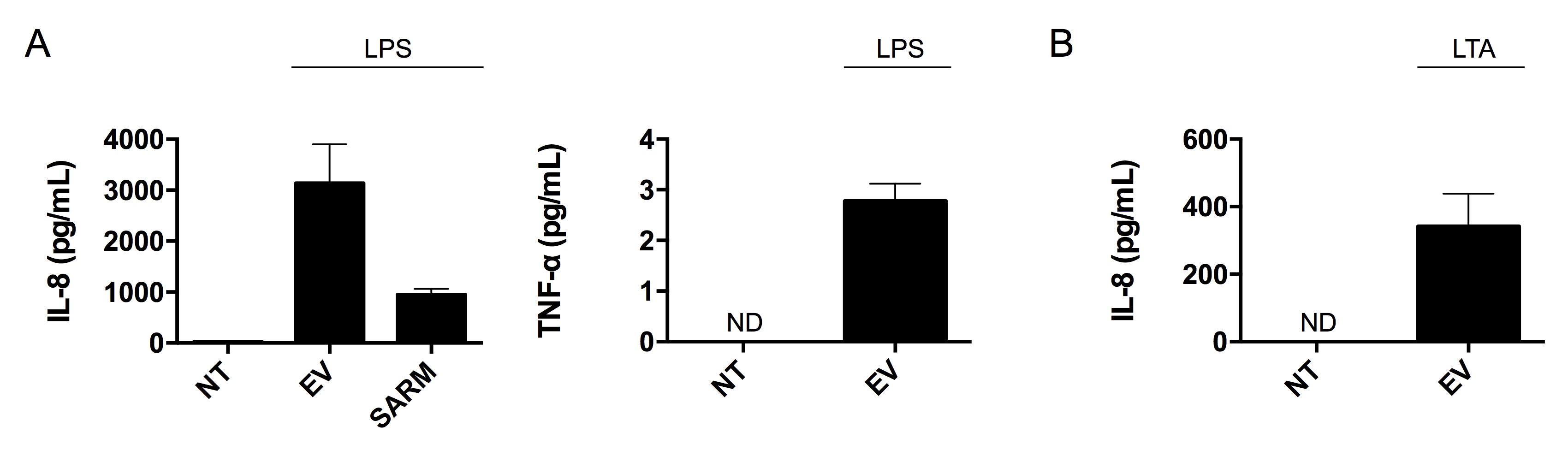

Supplement: S2 Fig — (A) HEK-TLR4 cells were transfected with 1000 ng of empty vector (EV) or a vector containing the gene encoding SARM, a known inhibitor of TLR-signalling. 24 hours post-transfection, cells were challenged with LPS (0.1 μg/mL) and the production of inflammatory cytokines, TNFα and IL-8, were assessed by ELISA 24 hours later. Background levels of cytokines produced by non-transfected (NT) cells without LPS-stimulation were also assessed. Bars represent mean values of three independent experiments. Error bars, SD of triplicates. ND, not detected. (B) HEK-TLR2 cells were transfected with 1000 ng of empty vector (EV), followed by assessment of IL-8 content in the culture supernatant by ELISA after an additional 24 hours. Background levels of IL-8 produced by non-transfected (NT) cells without LTA-stimulation were also assessed. Bars represent mean values of three independent experiments. Error bars, SD of triplicates. ND, not detected. (TIFF) [file pone.0158575.s002.tiff]
